# A transcription factor 7-like 1-lipocalin 2 axis in the differentiation of keratinocytes

**DOI:** 10.1038/cddis.2016.152

**Published:** 2016-06-02

**Authors:** Y Liu, H Cheng, S Xiao, Y Xia

**Affiliations:** 1 Department of Dermatology, The Second Affiliated Hospital, School of Medicine, Xi'an Jiaotong University, Xi'an, China; 2 Department of Medicine, The Second Affiliated Hospital, School of Medicine, Xi'an Jiaotong University, Xi'an, China

In epidermis, tightly junctional keratinocytes in the granular layer and fully differentiated keratinocytes in the cornified layer together construct an effective barrier that prevents undue water loss, at the same time, protecting against a variety of insults. This function is one of the major purposes of the epidermal dynamic balance, which includes a precisely regulated process of keratinocyte proliferation, terminal differentiation, apoptosis, and shedding.^1^ In normal skin, such homeostasis is delicately maintained by cell cycle checkpoints and multiple cytokines. However, this homeostatic balance is dysregulated under pathological conditions such as psoriasis, warts, and squamous cell carcinoma (SCC). Dysregulated differentiation is usually characterized by abnormal marker expression in keratinocytes and parakeratosis in epidermis.^2^

One important marker in dysregulated keratinocyte differentiation is the lipocalin 2 (LCN2, or neutrophil gelatinase-associated lipocalin), which preferentially overexpresses in keratinocytes with abnormal differentiation or some skin diseases with parakeratotic epidermis.^3^ Over the years, many reports have shown that LCN2 is involved in cell differentiation by multiple mechanisms such as inhibiting nuclear factor κB pathway, activating Met/focal adhesion kinase cascade, upregulating mesenchymal markers, and downregulating epithelial marker E-cadherin.^4^ However, all these studies focused on the downstream signals which are regulated by LCN2 while neglected the mechanism of the upstream signals modulating LCN2, especially in skin diseases associated with dysregulated differentiation of keratinocytes.

Earlier study has implicated that LCN2 may be a critical downstream target of transcription factor 7-like 1 (Tcf7l1, also known as the high-mobility group box transcription factor Tcf3), which together with LCN2 promotes cell migration and skin wound healing.^5^ Tcf7l1 is an essential transcription factor that controls both embryonic and adult skin stem cell functions.^6^ Miao *et al.*^5^ found that Tcf7l1 promotes keratinocyte motility and responses to wounded skin repair through upregulating LCN2 expression. Actually, the differentiation of keratinocytes is precisely manipulated during wound repair.^6^ Therefore, the Tcf7l1-mediated LCN2 signals participate in the process of keratinocyte differentiation. Other evidences showed that the activation of Tcf7l1 is found in cutaneous keratinocytes with a poor differentiation status.^7, 8^ These findings supported a speculation that a Tcf7l1–LCN2 axis contribute to the dysregulation of keratinocytes differentiation. However, apart from the diverse functions of Tcf7l1 and LCN2, the necessity and mechanism of Tcf7l1 as an upstream regulator for LCN2 to bring about dysregulated keratinocyte differentiation remained unclear.

In a recent study published in *Cell Death Discovery*, Xu *et al.*^4^ used three kinds of cell models to investigate the interplay between Tcf7l1 and LCN2 at perturbation of keratinocytes differentiation including human foreskin keratinocytes (HFKs) with calcium stimuli, human papillomavirus type 16 E6/E7-transfected HFKs, and human SCC-13 cells. When compared with normal HFKs, these three cells displayed increased expression of Tcf7l1 and LCN2 and decreased expression of involucrin and loricrin, two specific differentiation markers. Moreover, Tcf7l1 depletion in HFKs abrogated the effects of calcium stimuli on cell differentiation by decreasing LCN2 but increasing involucrin and loricrin expression. Besides, LCN2 inhibition enhanced both involucrin and loricrin levels in Tcf7l1-overexpressing HFKs while had no effect on the expression of Tcf7l1 mRNA. Obviously, LCN2 meditated the Tcf7l1 function in keratinocyte differentiation. Given that LCN2 can form complex with either matrix metalloproteinase (MMP)-2 or MMP-9, which are also suggested to be functional in regulating cell differentiation,^9, 10^ Xu *et al.*^4^ had also investigated the role of MMP-2 and MMP-9 in dysregulated differentiation of keratinocytes. The results showed that upregulation of MMP-2 predominated in Tcf7l1-overexpressing HFKs. Then, MMP-2 depletion was embarked. Consequently, LCN2 production was reduced significantly while involucrin and loricrin expression increased. In addition, it revealed that the lower differentiation grade corresponded to the more activated Tcf7l1 and LCN2. These results were mirrored by the tissular analysis of some skin diseases that have dysregulated epidermal differentiation such as psoriasis, warts, and so on. In general, the current models indicated the important role of Tcf7l1−MMP-2−LCN2 axis in managing skin diseases with dysregulated keratinocyte differentiation (Figure 1).

The responses to Tcf7l1–LCN2 axis activation, which may be notable in either normal or dysregulated keratinocyte differentiation, are the expression of differentiation markers and the decrease of cell apoptosis. In healthy skin, the normal process of differentiation involves expressing appropriate levels of molecular or morphological markers of the terminally differentiated state and accurately regulating the process of keratinocyte proliferation, terminal differentiation, and apoptosis. On the contrary, in aberrant keratinocyte differentiation regulated by Tcf7l1–LCN2 axis, the keratinocytes fail to express appropriate differentiation markers such as involucrin, loricrin, caspase-14, and keratin-1.^11^ Besides, there is a disorder of keratinocyte proliferation and apoptosis.^12^ Hence, Tcf7l1–LCN2 signaling in wound repair or other skin diseases is likely to disturb terminal differentiation and maintain cells in an abnormal differentiation state. Overall, these findings indicate that Tcf7l1–LCN2 axis may have double-edged effects on regulation of keratinocyte differentiation.

Inhibiting the Tcf7l1–LCN2 axis as a therapeutic method for managing skin diseases with pathological epidermis state comes with reversion of the abnormal differentiation of keratinocytes. In that work, both type and stage of dysregulated differentiation matter importantly. Moreover, agents that can specifically target the type or stage while shunning away from the potential adverse effects on normal keratinocyte migration and differentiation are highly valuable. Getting down to the journey of translating Tcf7l1–LCN2 axis from laboratory bench to bedside, those responses in keratinocytes to Tcf7l1–LCN2 activation during dysregulated differentiation may come in handy.

## Figures and Tables

**Figure 1:**
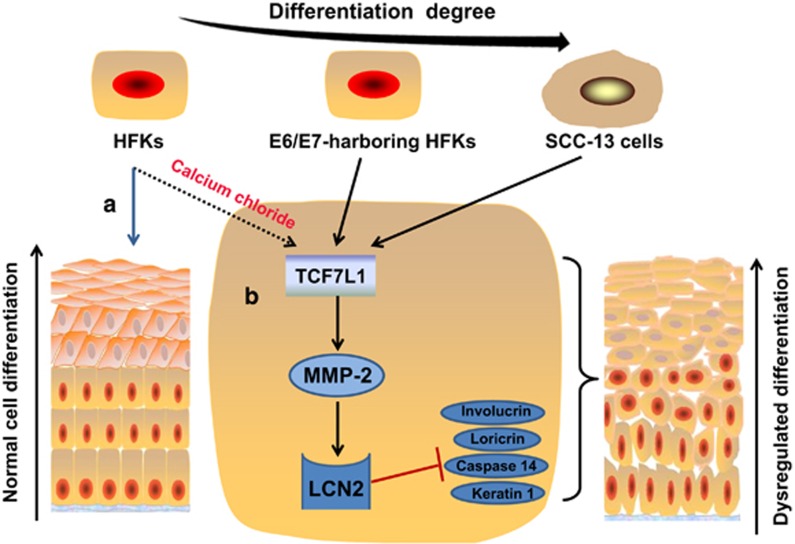
The roles of Tcf7l1–LCN2 axis in keratinocyte differentiation. (**a**) In healthy skin, epidermal differentiation is a complex process with keratinocyte developing from a proliferative cell type in the basal cell layer of the epidermis into flattened, dead cells in the outermost layer, the stratum corneum (**b**) In the HFKs with calcium stimuli, E6/E7-transfected HFKs or SCC-13 cells, differentiation is dysregulated by the upregulation of Tcf7l1, which further leads to an increase in LCN2 by upregulating MMP-2. Meanwhile, LCN2 can interact with MMP-2 to decrease the expression of involucrin, loricrin, caspase-14, and keratin-1. Some skin diseases featuring epidermal parakeratosis follow such rule in keratinocyte differentiation
